# “Common sense is not always enough”: preschool educators’ perspectives on young children’s engagement with bodies and weight

**DOI:** 10.1080/17482631.2026.2760666

**Published:** 2026-11-06

**Authors:** My Sjunnestrand, Nicklas Neuman, Ximena Ramos Salas, Kajsa Järvholm, Karin Eli, Paulina Nowicka

**Affiliations:** a Department of Food Studies, Nutrition and Dietetics, Uppsala University, Uppsala, Sweden; b Replica Communications, Kristianstad, Sweden; c Bias 180, Dundas, Canada; d Department of Psychology, Lund University, Lund, Sweden; e Warwick Medical School, University of Warwick, Coventry, United Kingdom

**Keywords:** Childhood obesity, weight bias, weight stigma, educational settings, early childhood qualitative research, parents, families

## Abstract

**Purpose:**

The study explored how preschool educators perceive and respond to young children’s interactions around bodies and weight, to understand educators’ readiness to mitigate early weight bias and stigma.

**Methods:**

Semi-structured interviews were conducted with Swedish preschool educators (*n* = 23, all female, mean experience = 18.7 years, 78% with a university degree). Interviews were transcribed verbatim and analysed using reflexive thematic analysis.

**Results:**

Three themes were identified. *Neutral understanding of bodies* included *Friendship beyond body size* and *Innocent curiosity*, capturing educators’ views that children’s interactions around bodies and weight had limited impact on social relationships. *The emergence of weight bias* included *Weight stigma with intention* and *Feeling and being different.* Educators described deliberate stigmatising behaviours and signs of children internalising weight stigma. *Meanings and responsibilities around children’s bodies* included *Challenging early weight labelling, Extending weight stigma to parents,* and *Sensitive topics with limited support.* Educators expressed that labelling children as having obesity might cause psychological harm; others recognised obesity as a concern tied to parental practices. Educators described feeling responsible to model inclusive practices, yet lacked structured guidance.

**Conclusions:**

Training and support could help educators address discussions around bodies and weight sensitively, foster positive body image, and reduce weight bias in preschool settings.

## Background

Preschools are formative environments where young children begin to make sense of their own and others’ bodies. Experiences and interactions in preschool settings may influence children’s emerging body image and their relationship with food and eating (Eli et al., 2014; Tatangelo et al., 2016), and the ways in which they are framed are therefore crucial. In preschool settings, educators play a key role in addressing children’s engagement with these topics. Yet, little is known about how preschool educators experience and respond to such discussions in practice.

Body size and weight are sensitive issues, and interactions related to these matters in preschool settings may influence children’s emerging body image and their perceptions and attitudes toward others (Paxton & Damiano, 2017). Weight bias (i.e., negative weight-related attitudes) is evident in early childhood and has been observed in children as young as three years of age (Bensley et al., 2023). By the time children reach primary school, these attitudes may contribute to teasing and social exclusion of children living with overweight and obesity (van Geel et al., 2014). Given the strong influence of peers in young children’s developing body image (Dohnt & Tiggemann, 2006; Paxton & Damiano, 2017), such attitudes may have implications for children’s understanding of bodies and weight. Around the preschool years, children also begin to develop theory of mind—the ability to understand that others have thoughts, feelings, and perspectives that may differ from their own (Herrmann et al., 2007). With this development comes children’s capacity to compare themselves to others (Brummelman & Thomaes, 2017) and to consider how they may be viewed by others (Asaba & Gweon, 2022). As children become increasingly aware of how others perceive them, experiences of weight bias may be internalised over time (Fields et al., 2021; Gmeiner & Warschburger, 2020).

In this context, educators play a particularly important role, as they can influence how body-related talk is framed, which messages are emphasised, and how weight concerns are addressed. Thus, the way educators respond to children’s interactions about bodies and weight may either mitigate or reinforce expressions of weight bias. While no studies have explored preschool educators’ perspectives on this, studies involving school staff working with older children have shown that conversations about bodies and weight in school settings are often perceived as emotionally demanding and difficult to manage due to their sensitive nature (Chisholm et al., 2022; Coupe et al., 2022; Howard-Drake & Halliday, 2016; Thorstensson et al., 2018). For example, a qualitative study in Sweden found that elementary school nurses were concerned that discussing overweight and obesity with children and their parents might be perceived as offensive (Thorstensson et al., 2018). This reluctance may stem from concerns of unintentionally reinforcing weight stigma—harmful stereotypes that lead to social devaluation of people because of their body weight or size (Puhl & Heuer, 2009)—which has been identified as a barrier to addressing children’s weight and health behaviours (Chisholm et al., 2022). At the same time, educators may themselves hold weight bias (Nutter et al., 2019). Yet, it remains unclear whether and how weight bias manifests in preschool settings and how educators respond to it.

Sweden provides a particularly relevant context for examining young children’s interactions around bodies and weight, given that 90% of children aged 1–5 years are enrolled in preschools, and therefore experience consistent and substantive interactions with their peers (Swedish National Agency for Education, 2025). From age three, children are entitled to 15 hours per week of free child care, with additional hours subsidised through an income-based fee (Swedish National Agency for Education, n.d). This fee covers all meals provided at preschools, all of which should be nutritionally balanced and evenly distributed throughout the day (Swedish National Agency for Education, 2025). Preschool staff includes both preschool teachers, who hold a 3.5-year university degree, and child minders with upper secondary education in child care (Swedish National Agency for Education, 2025), henceforth collectively referred to as “educators.” The educators follow a national curriculum that emphasises children’s development, health, and well-being. The curriculum also states that all preschool staff should foster human dignity and solidarity, ensuring that no child is subjected to discrimination (Swedish National Agency for Education, 2019). Thus, educators are expected to create a supportive environment that addresses sensitive topics in an inclusive and empathetic manner.

As many young children spend considerable time in preschool, and preschool educators are uniquely positioned to observe and influence children’s everyday interactions, educators’ perspectives offer valuable insights into how interactions surrounding bodies and weight take shape in these environments. Currently, there is a substantial gap in the literature concerning educators’ perspectives on children’s interactions around bodies and weight in preschool settings, as existing research has primarily focused on older children. The aim of this study is therefore to explore how preschool educators perceive and respond to young children’s interactions around bodies and weight, in order to understand their readiness to mitigate the early formation of weight bias and stigma.

## Materials and methods

### Recruitment and participants

This qualitative study is based on individual, semi-structured interviews with preschool educators (*n* = 23) in Sweden. The sample size was consistent with the model of “information power”, in which the required number of participants was influenced by the study aim, sample specificity, richness of the data and analysis strategy (Malterud et al., 2016). For example, our study’s relatively narrow aim, the participating educators’ experiential specificity in relation to the phenomenon under investigation, and the rich data generated by the interviews supported a smaller sample size. However, as our analysis focused on identifying common themes across educators’ accounts, rather than conducting an in-depth analysis of a few individual interviews, this suggested a need for a larger sample size. Thus, the sample of 23 educators provided the information power required to address the study aim.

Eligible participants were preschool educators fluent in Swedish and with at least 1 year of experience working with preschool children. While educators of any gender were eligible for the study, only women volunteered for interviews (Table I). This is consistent with the female-dominated preschool workforce in Sweden, where approximately 96% are women (Swedish National Agency for Education, 2025). Given this, no additional efforts were made to recruit other genders.

Recruitment was carried out using purposive sampling (*n* = 21), complemented by snowballing (*n* = 2). Educators were initially contacted via emails to preschool directors, advertisements on social media, personal networks, or direct visits to preschools. Interested individuals received detailed study information via email and were asked to provide written informed consent prior to participation. All informed consent sheets were collected digitally, except for one that was returned by mail in line with the participant’s preference. Once consent was obtained, interviews were scheduled at a time and place suitable for each participant. Of 25 expressions of interest, two declined due to time constraints. Participant characteristics are presented in Table I. Data collection took place between March and May 2025.

**Table I.** Characteristics of participating educators.

Educators (*n* = 23)	
	**Mean (SD)**
**Age (years)**	46.9 (10.9)
Experience as preschool educator	18.7 (12.1)
**Country of birth**	***n* (%)**
Born in Sweden	17 (74)
Born outside Sweden	6 (26)
**Gender**	
Female	23 (100)
**Education**	
Upper secondary school	5 (22)
College/University	18 (78)
**Position**	
Preschool teacher	16 (70)
Child minder	6 (26)
Other	1 (4)

### Data collection and analysis

#### Semi-structured interviews

We used semi-structured interviews with open-ended questions, which allowed us to probe educators’ experiences flexibly and follow up on insights as they emerged (Adams, 2015). The interviews were conducted by MS (*n* = 17), a doctoral student specialising in weight stigma in early childhood, and by an undergraduate student (*n* = 6) pursuing a B.Sc. in nutrition, both supervised by MS and PN. Most interviews were conducted via a videoconferencing platform (Zoom), except for two telephone interviews, in line with participants’ preferences. The interviews followed an interview guide (Appendix 1) developed by MS in collaboration with the co-authors, all of whom are experienced in childhood obesity and qualitative research. Questions were open-ended, neutrally worded, and explored how children of different body sizes play and interact (e.g., *“Have you heard the children in the preschool/kindergarten group comment on someone else's weight or body shape? Can you give examples?”*) and how preschool educators respond to these interactions (e.g., *“How did you handle it?”).* Follow-up questions were asked depending on the participants’ responses.

The guide was informed by previous research on children’s interactions around body-related talk and weight bias, our study’s aims, and the research team’s experience in childhood obesity, weight stigma, and qualitative research. Based on a brief literature review, MS developed the first draft of the interview guide. The draft was circulated among the broader research team, who provided feedback. Questions were revised through team discussions to align with the study aims and to facilitate open, non-leading dialogue. The guide was then pilot tested with two preschool educators to assess the clarity and relevance of the questions, resulting in no further changes. The participants in the pilot test were not included in the final sample. Interviews lasted between 26 and 59 minutes (mean = 43 minutes), and only the interviewer and the educator were present. The interviewers were not acquainted with any participant before the study started. All sessions were audio-recorded and transcribed verbatim. Some interviews (*n* = 6) were transcribed manually by the undergraduate student, and the remaining (*n* = 17) were transcribed using a transcription feature integrated into Microsoft Word, accessed through the university’s secure systems, followed by manual corrections by MS.

#### Thematic analysis

MS analysed all interview transcripts using reflexive thematic analysis (Braun & Clarke, 2022). The analysis began with MS reading and re-reading all transcripts, during which analytic memos were recorded. Subsequently, key data segments relevant to the research questions were transferred to an Excel spreadsheet, and systematically coded using an inductive approach. When transferring these data segments, we paid careful attention to preserving the relevant context, denoting it in an adjacent column and/or including longer extracts to ensure that all quotes were interpreted with appropriate nuance. The codes were generated from the data itself, rather than from a pre-defined coding framework (Braun & Clarke, 2006). Once all transcripts were coded, potential themes and subthemes were identified. This process was facilitated by creating a thematic map in the software Miro (2024), where codes were organised and grouped according to the initial themes and subthemes. The themes were then reviewed and further developed through an iterative process that assessed their robustness and distinctiveness, leading to some themes being merged and others being divided into separate themes. The analysis was further refined through iterative discussion among all co-authors, resulting in the restructuring, defining, and renaming of certain themes and subthemes. Once all themes had been defined and deemed coherent, we draughted the Results section, supporting our interpretations with illustrative quotes. Table II provides an example of how codes and themes were derived from the data.

**Table II.** Example of coding and theme development.

Data segment	Code	Subtheme	Theme
*They chose to opt out themselves… that they feel, “I can’t do this”, maybe because “I’m heavier” or “I don’t have the stamina” or whatever it is… “I can’t get up on this rock,” or “I can’t crawl under this stick because it’s not going to work,” and then, “I don’t want to join in,” and so they walk away.*	Opting out of play	Feeling and being different	The emergence of weight bias
*It’s a very innocent kind of comparing, it’s about discovering their bodies […] like, “Oh, you have a big belly, I have a small belly, okay, now… now let’s go play something else.*	Neutral body comments	Innocent curiosity	Neutral understanding of bodies
*How are you eating at home then? How did it come to this? […] I know there was a lot of TV watching and candy, at home you let them do whatever, and then I’m supposed to … I have to deal with it [laughs]. (Educator code 695)*	Parental influence on child’s weight	Extending weight stigma to parents	Meanings and responsibilities around children’s bodies

### Reflexivity and rigour

The multidisciplinary research team brings together complementary expertise in paediatric obesity, the sociology of food, public health, child psychology, and medical anthropology. This breadth of knowledge provided a strong foundation for analysing the data and for interpreting educators’ reflections through multiple lenses of child development, health, and culture. For example, expertise in paediatric obesity and public health informed the study’s focus on early weight-related attitudes, while perspectives from medical anthropology and the sociology of food contributed to the interpretation of educators’ talk as situated within broader social and moral meanings of eating and the body. The psychological perspective further supported a nuanced understanding of educators’ emotional and relational responses in their everyday interactions with children. The collective expertise of the co-authors also influenced study design and interpretation. For instance, it informed our decision to include questions on early weight-related attitudes, and our interpretation of how weight stigma appeared in educators’ descriptions of children’s engagement with bodies and weight.

## Ethical statement

This study was conducted in accordance with the Declaration of Helsinki and was approved by the Swedish Ethical Review Authority (ID: 2024-07381-01). Informed consent was obtained from all participants.

## Results

The thematic analysis resulted in the development of three main themes, each encompassing two or three subthemes (Figure 1). Each theme is presented below, including illustrative quotes from educators, followed by a randomly assigned anonymized code.

**Figure 1.**
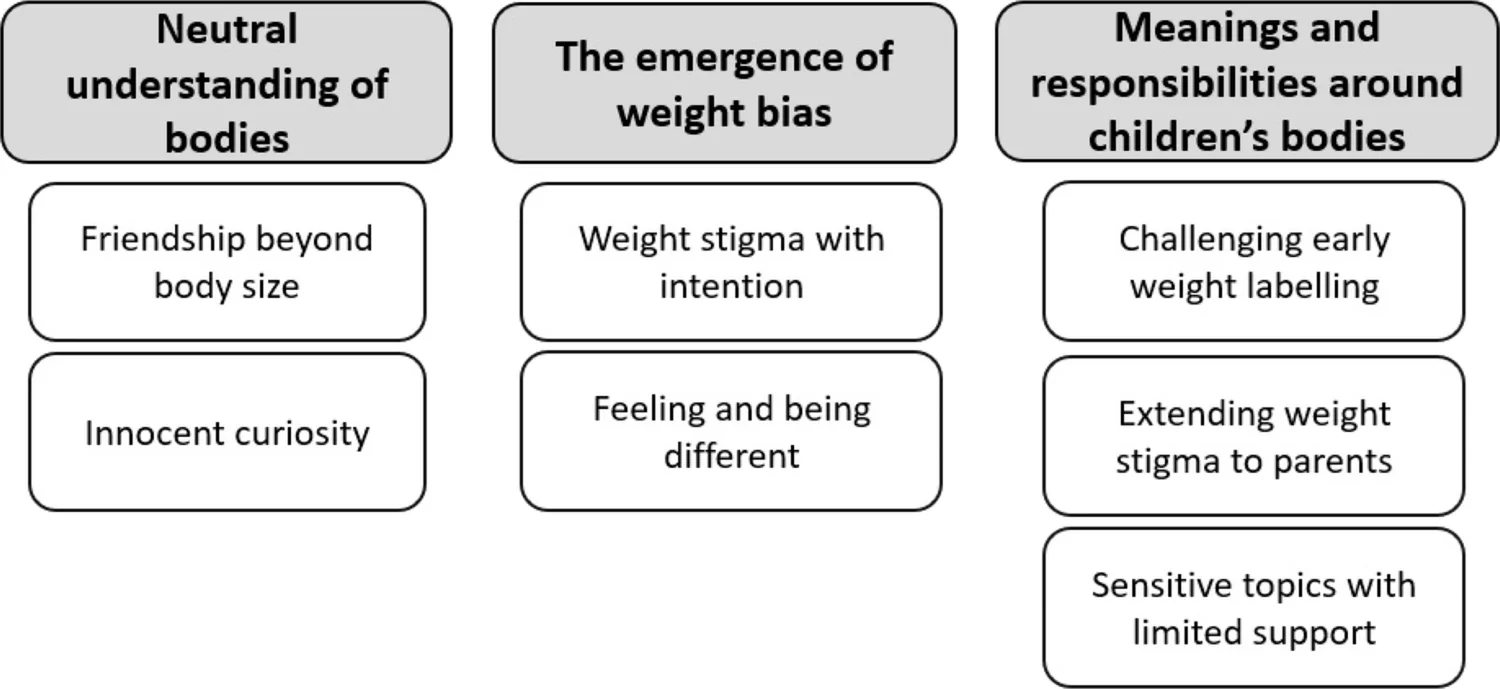
Identified themes and subthemes.

### Neutral understanding of bodies

This theme covers some educators’ accounts of how physical characteristics play a minor role in preschool children’s social relationships, which educators attributed to the children’s young age. It highlights how educators interpret children’s comments about body size as neutral expressions of curiosity, and how their responses range from validation to setting boundaries. The theme includes the subthemes *Friendship beyond body size* and *Innocent curiosity.*

#### Friendship beyond body size

Several educators reported they had never experienced children making negative comments about weight or excluding peers from play or other social activities based on body size. One educator noted this with relief:

Actually, that’s one of the nicest things about working in preschool - I feel that children are … so free from prejudice, that everyone is like, “As long as you’re kind, it doesn’t matter.” I think it’s wonderful. We should learn more from children. (Educator code 196)

According to some educators, however, children sometimes described peers as “small” or “large”, though this typically referred to age or height. They reported that children with larger bodies were often perceived as strong by their peers, and a few educators observed that being “bigger” than peers could be seen as a status symbol. Overall, educators stated that children tended to form friendships based on factors other than body size, such as gender, personality traits, and shared interests.

I don’t see any difference. … When you’re that young, it’s not about [body] ideals for me, it’s just about playing together. “Now I play with him because this is fun to play together. Next time I play with her because we enjoy this”, and so on. (Educator code 933)

The quote above reflects the educator’s view that preschool children are too young to have formed ideals about body size, suggesting that such ideas develop later in childhood. Several educators shared this perspective, expressing a strong belief that preschool children rarely reflect on physical appearance. School entry was described as a turning point, when children begin to notice differences in physical appearance, with weight-related teasing, bullying, and social exclusion based on body size becoming more common. Participants associated this change with the growing influence of weight bias as children grow older, which they attributed to parents, social media, and society at large.

#### Innocent curiosity

While several educators reported rarely noticing weight-related comments among preschool children, others indicated that such remarks did occur. These educators described instances in which children commented on or asked questions about others’ bodies—for example, by making neutral statements about someone having a large bottom or asking if an adult was pregnant. Educators who had heard such comments often interpreted them as expressions of genuine curiosity, a way for the children to make sense of the world around them. One educator explained:

They might ask me, “[Name of educator], why do you have such a big belly?” But it’s like…? Well, I don’t know. […] “Yes, I look like this. I’m overweight. I know that.” … Should I say, “You know, that’s not nice to say”? Yes, but maybe the child thought so and wondered why. I don’t attach any negative or positive judgement to it, but I see it more as a question of curiosity from the children. Maybe it’s part of their development, trying to understand people’s differences. (Educator code 145)

The quote above reflects the educator’s rather uncomplicated approach to the child’s questions about her belly. By responding “Yes, I look like this”, the educator affirmed the child, treating it as an innocent question rather than something requiring correction. This approach was shared by other educators with similar experience; some would add that everyone looks different and that this is perfectly fine. In certain cases, particularly when children used the term “fat” to describe another child or adult, educators explained that commenting on others’ bodies is inappropriate. The latter approach appeared more common when the comments were directed to another person, for example, a peer or a parent, rather than the preschool educator herself, as illustrated by the following quote:

Then… we have a child who is very… big. And it’s this child who is very fond of food. And this child has also been told a few times [by children], “You’re so fat!” Um… He actually seems quite unbothered and kind of responds like, “Yes”, a bit like, “Yes, I am.” And then I’ve stepped in and said, “You know, we don’t need to talk about how we look. We all look different.” (Educator code 147)

Some educators also noted how children compared themselves to one another and observed differences between their bodies. In most cases, educators interpreted these comparisons as value-neutral statements, as described by this educator:

It’s a very innocent kind of comparing, it’s about discovering their bodies […] like, “Oh, you have a big belly, I have a small belly, okay, now… now let’s go play something else.” (Educator code 847)

In the quote above, the educator emphasised that the comparison was simply observational rather than judgmental. A common approach among educators when they heard such comments was to wait for the other child’s reaction and only interrupt if the other child became upset. Educators mentioned that this strategy was used to avoid projecting their own values or implying that one body shape is better than another.

### The emergence of weight bias

This theme reflects educators’ accounts of how children’s curiosity about different bodies can turn into stigmatising comments, teasing, and social exclusion. It illustrates how educators respond by fostering empathy and inclusion, while also observing the early emergence of body awareness among children. The theme encompasses the subthemes *Weight stigma with intention* and *Feeling and being different.*

#### Weight stigma with intention

Although most educators interpreted weight-related comments among children as an innocent exploration of differences, some reported instances where children made intentionally negative remarks about body size. In some cases, educators overheard these comments directly, while in others, they learned about them from parents who had been informed by their children. For example, one educator (code 249) recounted that a parent had reported their child being told by peers: *“You’re so fat … you’re not as pretty as us.”*

In most cases, educators understood these comments as words and phrases that children had picked up without fully grasping their meaning. However, a few educators described instances where such terms were deliberately used to tease or upset other children. These educators also recounted situations in which bigger children were excluded from play or other social activities by their peers. One educator, who identified hurtful comments and social exclusion as a significant problem during a particular period at her preschool, shared:

Then we have a girl who... is a bit bigger, and that’s been more difficult. Because girls are a bit harder to reach because they’re a bit sneakier, they sneak around it in different ways... “She can join in and draw just like the rest of you … how you look has nothing to do with it”, I said. […] But then we’ve had boys who’ve actually said things like “fatty” and “fathead”, you know, those kinds of... unpleasant words. (Educator code 385)

In this quote, the educator highlighted perceived gendered differences in how children engage in exclusion. Girls are described as being more indirect in their behaviour—“sneakier”—while boys are portrayed as more overt in their use of stigmatising terms. When such incidents occurred, a common pedagogical approach among educators was to evoke empathy by encouraging children to reflect on how their comments might affect others. The educator continued:

When you talk to them afterwards, it’s just like, “Well, I don’t mean it that way.” And we say, “No, we understand you didn’t, but it still hurts the other child. The other child gets a hole in their heart, and that’s… it’s hard, because it leaves a scar. You can’t see it, since it’s inside the other child.” (Educator code 385)

Referring to the behaviour as leaving a scar, the educator aimed to help the child understand that such remarks could have a lasting emotional impact. Other educators emphasised that everyone looks different and that each child is valuable just as they are, regardless of appearance. Some used pedagogical tools, such as storytelling or theatre, in which they role-played real situations that had occurred at the preschool and encouraged children to reflect on their behaviours afterward, with the intention of helping children put themselves in the other child’s situation.

#### Feeling and being different

While educators described instances where children were excluded by their peers, they also observed that children with larger bodies sometimes excluded themselves from play. Specifically, educators noted that these children often chose not to participate in social activities involving active play, such as races or climbing. One educator shared:

They chose to opt out themselves… that they feel, “I can’t do this”, maybe because “I’m heavier” or “I don’t have the stamina” or whatever it is… “I can’t get up on this rock,” or “I can’t crawl under this stick because it’s not going to work,” and then, “I don’t want to join in,” and so they walk away. (Educator code 287)

Educators reported that when they noticed children wanting to withdraw from physically demanding activities, they would encourage or assist them. For example, they might hold a child’s hand during races and offer to collaborate, raise a stick to help the child crawl under, or provide verbal encouragement. One educator reflected on her strategy:

They want to participate on the same terms, but they don’t always manage it, and then we need to support them. We can also find other activities, but most of the time it’s about pushing them just enough so they don’t lose their confidence. (Educator code 847)

This quote illustrates the educators’ sense of responsibility to protect children’s confidence—a concern shared by other educators. Educators also described observing that as children grew older, they appeared to develop a judgmental and sometimes self-stigmatising awareness of their own bodies, with children with larger bodies sometimes commenting on their size. For example, one educator recalled an instance when she visited a public swimming pool together with the oldest children in the preschool (5 years old), and a child noticed another wearing a bikini:

So, she [the child] looked and said, “You know what? I’d like to have one of those [a bikini], but I don’t think it suits my body, because I’m big.” (Educator code 379)

When the educator heard this, she reassured the child that bodies come in many shapes and sizes and that there are clothes that fit everyone. Other educators reported using the same approach and emphasised that they actively avoided attaching any judgement to such comments. Some educators also mentioned engaging in a general practice of promoting body acceptance at the preschool. This included deliberately speaking positively about themselves in front of the children or encouraging children to make positive statements about themselves through affirmations.

### Meanings and responsibilities around children’s bodies

This theme highlights educators’ roles in shaping children’s early understanding of bodies and weight. It illustrates how they express a strong sense of responsibility to shield children from potentially harmful experiences, yet often lack structured guidance. The theme involves the subthemes *Challenging early weight labelling, Extending weight stigma to parents,* and *Sensitive topics with limited support.*

#### Challenging early weight labelling

As educators reflected on children’s body size, some expressed concern about labelling children as having overweight or obesity, as they believed it could be harmful to place too much emphasis on weight at such a young age. Most educators refrained from using the term “overweight” and instead described children with larger bodies using euphemisms such as “a little bigger than the peers” or “sturdy”, as illustrated by the following quote:

I mean, she was big, this girl, but definitely not over[weight], or, if you even use the word, “fat.” She was sturdy, yes, and a bit… and she was taller than the other kids her age, so she was a… big girl, but… And definitely not slim, I guess I can’t really say that. (Educator code 471)

Some educators questioned the use of Body Mass Index (BMI) for young children, arguing that overweight and obesity are not major concerns in preschool and that a certain degree of “chubbiness” is typical at this age. One educator remarked: *“I think it’s completely crazy that… preschool children should lose weight and go on a diet”* (Educator code 490). Educators emphasised that body sizes vary naturally within a population and believed that children who appeared bigger than their peers would grow into their bodies if given sufficient opportunities for physical activity. At times, this perspective led some educators to feel sceptical when informed that a child had overweight. One educator shared an example:

We had a case where a child had a check-up at the child health centre at age four, [the child] came back and then the parents say, “Yes, they said she’s overweight” […] We thought it was really strange that this child was overweight, I mean, that she would be overweight, we just didn’t understand where it came from. And we talked among ourselves, “What? How can anyone say that about this little child?” (Educator code 287)

The quote above reveals the educator’s disbelief concerning the child’s weight status, and indicates a view that the child is “too young” to be classified in the overweight category. By referring to her as “this little child,” the educator demonstrates a sense of responsibility to protect the child and implies that such a label may be inappropriate at this young age. In some cases, educators suspected that parents, rather than healthcare professionals, claimed their child had overweight despite a BMI within the normal range, due to concerns about future weight gain.

#### Extending weight stigma to parents

While some educators expressed disbelief or scepticism toward applying BMI and higher weight status to children, others recognised childhood overweight and obesity as a concern. One educator expressed concern in particularly strong terms, describing severe overweight among young children as “*a form of child abuse*” (code 865). Educators often attributed children’s overweight to excessive screen time, sedentary behaviours, and unhealthy eating habits at home. At times, these perceptions created discrepancies between educators’ and parents’ views on the importance of initiating measures at the preschool. Specifically, educators expressed a reluctance to limit a child’s food intake in response to parental concerns. An educator explained:

How are you eating at home then? How did it come to this? […] I know there was a lot of TV watching and candy, at home you let them do whatever, and then I’m supposed to … I have to deal with it [laughs]. (Educator code 695)

The quote above reflects a tendency to attribute the child’s overweight to parental practices. The educator’s reference to screen time and candy consumption suggests a view that parents lack control and should assume greater responsibility for their child’s lifestyle habits. Other educators shared this perception but distinguished between children whose weight gain they attributed to unhealthy habits and those whose body size they linked to a diagnosis, medical condition, or lack of satiety cues. For example, some educators noted that certain children appeared to have an unusually large appetite, which they perceived as indicative of a medical issue. In these cases, educators reported greater motivation to support the child’s food intake.

#### Sensitive topics with limited support

Several educators reflected on the lack of guidance and support in preschools regarding issues related to children’s weight and bodies. For example, this involved restricting a child’s food intake without drawing attention to the child, or how to respond when children made weight-related comments about themselves or others. “*I haven’t received any [support] in my 13 years, ever. So that’s really remarkable*” (Educator code 847). This lack of support was seen as particularly challenging during the early stages of an educator’s career, when their practical experience was limited. Nonetheless, almost all educators, regardless of experience, expressed a desire for additional support to handle these situations sensitively and confidently. An educator shared her view:

I think common sense alone isn’t enough when it comes to these issues in preschool. There are many people working here, with different levels of experience. We have, like, substitutes who say things like, “Oh my gosh, you’re eating so much! You’re going to get really fat!” … You just want to crawl into bed when you hear stuff like that. (Educator code 147)

This educator’s reflection illustrates how the absence of clear guidance and training may contribute to staff conveying stigmatising attitudes. Others described similar experiences, emphasising the importance of carefully choosing words when speaking to and around children. They expressed a strong sense of responsibility for providing a safe and supportive preschool environment, and for helping children feel valued regardless of their appearance. Educators described children as highly attentive, even to comments that adults might assume go unnoticed. They acknowledged their own role in shaping children’s developing ideals of health and body size:

And my god, the little kids are like little satellite dishes too, around three or four years old when their understanding develops. They hear everything. So, you have to be careful. (Educator code 695)

Nevertheless, most educators considered caregivers, older siblings, and the media to be the primary influences shaping children’s body image and perception of body size.

## Discussion

Our study is among the first to explore preschool educators’ perspectives on young children’s engagement with bodies and weight, while highlighting educators’ potential contribution to or mitigation of children’s weight bias and stigma. We found contrasting perspectives in educator’s accounts. Several educators perceived children’s engagements with weight and size as neutral explorations of differences with limited impact on children’s social relationships. However, some described instances of intentional weight stigma, along with early signs that children may already be internalising negative, weight-related messages. A few educators raised concerns about labelling children as having obesity; others acknowledged childhood obesity as a health concern, and some attributed it to parental practices. Across these accounts, educators expressed a strong responsibility to shield children from harmful experiences, yet reported lacking structured guidance.

According to educators, children with higher weights were occasionally subjected to negative weight-related comments and teased or excluded by their peers. While previous research indicates that weight bias may emerge as early as the preschool years (Bensley et al., 2023), our study provides new insights into the social implications of this bias for young children. At the same time, educators described how children talk about bodies in neutral, non-judgmental ways, indicating that weight bias among young children may not be inevitable but shaped by the social and cultural messages they encounter in everyday life. These findings are consistent with theories of early social categorisation, which suggest that while young children naturally group people to make sense of their world, the meanings attached to these groups are shaped by adult labels (Bigler & Liben, 2006; Bigler, 2013). As children’s theory of mind develops, they gradually gain the ability to infer others’ perspectives and interpret social norms (Herrmann et al., 2007). Meeting educators who attribute overweight to inadequate parental practices may shape how a child perceives themselves and their family background.

From a prevention perspective, early childhood represents a critical period for addressing the development of weight bias. Previously, schools have been identified as a key setting for weight bias reduction interventions (Ramos Salas et al., 2017). Our findings suggest that expanding such efforts to preschools may be valuable. While evidence on interventions specifically targeting weight bias remains scarce, research on other forms of bias, including racial bias and bias towards individuals with intellectual disabilities, indicates that interventions implemented during the preschool years may effectively reduce young children’s prejudices (McManus et al., 2021; Slobodin et al., 2023). In Sweden, education about equal value is already embedded in the preschool curriculum (Swedish National Agency for Education, 2019), allowing issues related to bodies and weight to be integrated with existing work on inclusivity. One possible approach may be to develop age-appropriate educational materials, such as storybooks that represent diverse bodies in an inclusive way (Itzoe & Frasso, 2021). Such efforts are important, as exposure to weight bias may become internalised by children and contribute to the development of a negative body image (Foster et al., 2024). Importantly, work on body and weight inclusivity in preschools requires a distinction between natural variation in body size and obesity as a medical condition, since body size alone does not necessarily reflect health behaviours or health status. Conflating body size with health or health-related behaviours may unintentionally reinforce stigmatising beliefs. Thus, efforts to promote body acceptance and reduce weight stigma in early childhood should be integrated with efforts to support healthy weight development.

Furthermore, our findings suggest that, according to educators, young children may already be beginning to internalise weight bias. While some educators reported that children perceived those with larger bodies as strong, they also described how some children with higher weights avoided physically demanding activities or commented on their own body size. This indicates that these children may perceive themselves as less capable. These observations provide important new insights, as the internalisation of weight bias in young children remains understudied (Paxton & Damiano, 2017). Existing research shows that preschool-aged children may experience body dissatisfaction regardless of weight category (León et al., 2018; Tatangelo et al., 2016). However, while our study did not identify any clear perceptions of gender-based differences in children’s body dissatisfaction, studies among preschool-aged children in Canada and Spain found that girls were more likely to experience body dissatisfaction and prefer a thin body shape (León et al., 2021; Tremblay et al., 2011). This could potentially explain why parents may perceive boys as more resilient to negative weight talk, as shown in a prior study on family weight-related communication (Sjunnestrand et al., 2024). Taken together, the educators’ observations raise the hypothesis that attitudes toward body size may emerge early in childhood, highlighting the importance of addressing weight bias from a young age to prevent its internalisation and long-term consequences (Bigler, 2013).

While the interviewed educators made efforts not to stigmatise children with overweight or obesity, some expressed weight stigma towards these children’s parents, portraying them as “having failed” to manage their children’s weights. This aligns with research from a large community-based sample in the US, where parents of children with obesity were perceived as less effective and supportive (Lee et al., 2022). Qualitative studies further indicate that parents of children with overweight or obesity experience blame and guilt from others because of their child’s body size (Ek et al., 2020; Gorlick et al., 2020; Turner et al., 2012). These findings reflect socially prevalent ideas that hold parents responsible for their child’s weight. This is concerning, as children may absorb negative cues when they perceive adults attributing overweight to poor parenting or judging family practices. Importantly, even subtle non-verbal cues from adults may be noticed by children (Skinner et al., 2020).

Our findings showed that some educators did not perceive overweight and obesity as problematic among young children, instead expecting children to outgrow excess weight and viewing discussions of overweight and obesity at the preschool age as inappropriate. This is consistent with research on parental perceptions, which shows that parents often believe children will naturally grow into their weight (Musa et al., 2024; Trigwell et al., 2014). However, as childhood obesity is a chronic disease, this view conflicts with guidelines emphasising early detection and intervention (Hampl et al., 2023). While we recognise that educators are not responsible for diagnosing or treating obesity, they should be informed by healthcare providers and parents when a child has a medical diagnosis, so they can support the recommended care plans in preschools. Preschool is a key setting where children eat many of their daily meals, and in Sweden, meals eaten in educational settings are explicitly highlighted as occasions for learning. If educators do not recognise overweight or obesity as a concern or assume that addressing it is solely the parents’ responsibility, opportunities for early intervention may be missed.

### Implications for educational policy and practice

Drawing on accounts from Swedish preschool educators, our findings highlight the need for structured guidance and support to communicate sensitively about bodies and weight. These findings align with recommendations from previous research, emphasising that professionals who work with children, including educators, could benefit from formal education and ongoing training to reduce weight stigma (Tanas et al., 2022). As educators described feeling responsible for protecting children, this underscores the importance of building confidence and providing practical tools to discuss weight and bodies without reinforcing weight stigma. Relying solely on “common sense”, educators’ risk being misled, attributing children’s weight to poor parenting, assuming health behaviours such as eating and physical activity always reflect a child’s weight status, or ignoring complex factors linked with body weight and health. Training could therefore help educators avoid making assumptions about weight based on body size or health behaviours. Such training could be developed and implemented by institutional educational authorities, in collaboration with preschool providers.

While research on interventions to reduce weight stigma in educational settings remains scarce, a recent review on randomised controlled trials across different populations, including adults and children, identified several strategies that have shown promising results. These included, for example, educating participants about the complex nature of obesity, initiatives focusing on body acceptance, and empathy-inducing approaches (Wang et al., 2025). However, as the authors point out, the paucity of longitudinal studies conducted limits our understanding of the long-term effectiveness of such efforts. Thus, future studies should focus on identifying how the effects of such efforts can be sustained over time across diverse settings, including preschool settings.

In addition to training on weight stigma, educators could benefit from education on obesity so they are prepared to support children living with this chronic disease. The variation in educators’ approaches further underscores a role to play for evidence-based guidelines, particularly given that many educators lacked institutional support. Providing preschools with such guidelines could help structure how children’s bodies, health, and eating behaviours are addressed within preschool settings.

### Strengths and limitations

One strength of this study is the multidisciplinary expertise and broad experience of the authorship team, which contributed to the study’s rigour and was further enhanced by the collaborative analytic process. In addition, most educators who participated in this study held an academic degree as preschool teachers (78%) and had substantial experience working in preschools (mean 18.7 years). This is a strength, as it allowed us to capture the perspectives of professionals with both formal training and extensive practical experience. On the other hand, including educators with diverse educational backgrounds and experience levels could have added depth to the study. For example, interviewing less-experienced educators might have provided greater insight into how experience influences confidence and safety when addressing these sensitive topics. Moreover, including male educators could have offered additional insights; however, our sample reflects the female-dominated preschool workforce in Sweden, where 96% of educators are women (Swedish National Agency for Education, 2025). These sample characteristics should be considered when assessing the transferability of the findings to other preschool contexts. While the aim of this study was to examine educators’ perspectives, the findings are limited to this group and do not capture the experiences of children, parents, or preschool directors. Furthermore, this study relied on educators' retrospective self-reported accounts rather than direct observation of educator-child interactions, which limits our ability to draw causal conclusions about how educators' responses shape children's weight-related attitudes and behaviours.

Another potential limitation is the use of multiple interview modes, with a few conducted by telephone and the majority via videoconference. This may have affected the interaction between the interviewer and the participants and potentially the depth of the data generated. However, a recent scoping review found that while remote data collection may be shorter in duration, the data generated are similar in quality and depth to those collected in person (Roberts et al., 2025). Remote interviews also facilitated the recruitment of participants from different parts of Sweden. In addition, the use of two interviewers with different levels of experience in research and qualitative methods is another potential limitation, as it may have led to variation in the depth and richness of the data generated. At the same time, involving two interviewers enabled the workload to be shared and provided opportunities for discussion and the exchange of experiences during the data collection process.

As educators were asked to reflect on their experiences across all preschools where they had worked, this may have introduced a risk of recall bias, particularly given their extensive experience. In addition, as body, weight and health are sensitive issues, educators may have provided responses that aligned with what they assumed the interviewer would want to hear. We acknowledge these as additional limitations. Furthermore, given the study’s focus, the educators included were likely those with a particular interest in children’s engagement with bodies and weight. They may have been more aware of such issues; hence, perspectives from educators with less interest in or awareness of them may have been underrepresented. An ethnographic approach could offer first-hand insights into how weight-related stigma is expressed and managed in everyday practice, underscoring its value for future research.

## Conclusion

While educators generally perceived children’s interactions surrounding bodies and weight as value-free explorations of bodily differences, some accounts indicated early signs of weight bias and stigma. At the same time, the participating educators’ sense of responsibility to protect children from negative experiences contrasted with a lack of institutional support and guidance. These findings highlight the need for practical guidance on addressing these topics sensitively, as educators' responses may play a role in either reinforcing or mitigating weight stigma, according to educators' own accounts, potentially shaping children's developing body image. To support this, structured training could be offered to educators, along with the establishment and implementation of institutional guidelines. Such efforts have the potential to reduce weight bias in this setting and foster a positive body image among children, and we suggest that future research evaluate the effects of training and institutional guidance interventions.

## Supplementary Material

Supplementary Material Weight of Words Interview guide.pdf

## Data Availability

The transcripts/data from this qualitative study are not accessible due to confidentiality agreements made with the participants.
