# Supplementary material for: “Common sense is not always enough”: preschool educators’ perspectives on young children’s engagement with bodies and weight
Source: Int J Qual Stud Health Well-being. 2026 Nov 6;21(1):2760666. doi: 10.1080/17482631.2026.2760666 (PMC13757835; doi:10.1080/17482631.2026.2760666)
Supplement: Supplementary file 1 [file ZQHW_A_2760666_SM3059.pdf]

Interview guide – Interviews

1. What does a typical day at preschool/kindergarten look like?
    a. What does the preschool/kindergarten group look like?
    b. What do the meals look like over a day?

2. Describe a meal situation.
    a. What are the conversations usually about?
    b. Where do the children eat – at the table? Together?
    c. Is there any involvement in food preparation? If so, what and how?

3. Have you heard the children comment on the food itself or talk about what is "healthy" (good) or "unhealthy" (bad)?
    a. How did you engage with them?

4. How do you talk about food and hunger with the children?

5. Are there any guidelines or policies regarding how you should work with healthy eating habits in preschool/kindergarten?

6. What role do you think nutrition and eating play in children's physical growth?

7. What role does play and movement have in healthy development?

8. What is your experience from play situations when children with diverse body types interact and engage with each other during play?
    a. Do you feel that children with obesity are treated differently? In what way?

9. Are there situations where children with obesity are excluded from play or other activities?
    a. How do you handle it?

10. Have you heard the children in the preschool/kindergarten group comment on their own weight or body shape?
    a. In what way? In what situation?
    b. How did you handle it?
    c. Is there anything you would like to do differently next time?

11. Have you heard the children in the preschool/kindergarten group comment on someone else's weight or body shape?
    a. Can you give examples?
    b. How did you handle it?

12. What do you think affects the children's view of body and weight? Where do their perceptions come from?

13. What are your own thoughts about children's weight?

    a. What are your thoughts about the role of the teacher in relation to children’s weight and body size?

14. Do you have any experience of being asked to limit a child's food intake, for example on the advice of a dietician or guardian?
    a. How did you handle thjs?
    b. How did it feel?
    c. What was this experience like for you personally and professionally?

15. Have you received any support regarding how you can respond to questions about overweight and obesity and about body size and weight in general?
    a. If yes: Tell us what the support looked like. What was good and what can be done better?
    b. If no: Would you like support? What could that support have looked like?

16. Does the preschool/kindergarten have any kind of policy or strategies to deal with or prevent bullying?
    a. If yes: do these include weight-based bullying?

17. How can the preschool/kindergarten work to promote a healthy relationship with food, movement and body in children?

18. Is there anything you would like to add before we finish?
